# Nephrotic-range proteinuria and central nervous involvement in typical hemolytic uremic syndrome: a case report

**DOI:** 10.1186/s12882-020-01979-3

**Published:** 2020-07-31

**Authors:** Chuan Shi, Chao Li, Wei Ye, Wen-ling Ye, Ming-xi Li

**Affiliations:** 1 Department of Internal Medicine, Peking Union Medical College Hospital, Beijing, People’s Republic of China; 2 Department of Nephrology, Peking Union Medical College Hospital, Beijing, 100730 People’s Republic of China

**Keywords:** Hemolytic uremic syndrome, Nephrotic syndrome, Central nervous system

## Abstract

**Background:**

Hemolytic uremic syndrome (HUS), a common subtype of thrombotic microangiopathy (TMA), is characterized by microangiopathic hemolytic anemia, thrombocytopenia, and acute kidney injury. Shiga toxin-producing *Escherichia coli* infection is the most common cause of post-diarrheal HUS. Kidney and central nervous system are the primary target organs.

**Case presentation:**

A 64-year-old male presented with HUS following bloody diarrhea. Nephrotic-range proteinuria and hypoalbuminemia were present at the acute stage and renal histology revealed common TMA features. Neurological involvement presented as confusion and impaired cognitive function. Cranial magnetic resonance imaging demonstrated bilateral T2 hyperintensities in the brainstem and insula. The patient received plasma exchange and supportive care. Both the renal and neurological impairments were completely recovered 3 months after the onset.

**Conclusion:**

We report an adult patient presenting with nephrotic-range proteinuria and central nervous system involvement at the acute phase of post-diarrheal HUS. The reversibility of the organ damages might predict a favorable outcome.

## Background

Hemolytic uremic syndrome (HUS) is characterized by microangiopathic hemolytic anemia, thrombocytopenia, and acute kidney failure. Post-diarrheal HUS, mostly seen in pediatric age group, is associated with enteric infection with a Shiga toxin-producing *Escherichia coli* or Shigella dysenteria [1]. STEC-HUS may occur as outbreaks or sporadic cases. *E. coli* O157: H7 and O104: H4 have been identified as major pathogenic strains [2].

Renal injury is universally present in HUS, and approximately 40% of patients require renal replacement therapy [3]. Microscopic hematuria and subnephrotic proteinuria are common findings in urinalysis. Nephrotic-range proteinuria is seldom present in HUS.

Central nervous system (CNS) involvement, frequently seen in pediatric HUS cases, had been rarely reported among adults until the German STEC outbreak in 2011. Documented neurologic findings range from mild headaches to seizures, cognitive impairment, and coma [4, 5]. Symmetrical vasogenic edema within the brainstem and thalamus have been reported as the most prevalent imaging findings [4, 5]. We hereby present an adult case of post-diarrheal HUS with nephrotic range proteinuria and neurological manifestations.

## Case presentation

A 64-year-old male was admitted with confusion, acute kidney injury, and thrombocytopenia in November 2018. Before the admission, the patient had diarrhea accompanied by abdominal pain. On day 2, diarrhea became bloody, and the patient received gentamycin from a local clinic. Stool culture or Shiga toxin test was not performed during the course of diarrhea. Although gastrointestinal symptoms resolved within a week, he developed confusion, dark urine, jaundice, and blurred vision 11 days after the onset of diarrhea, and was referred to our medical center on day 16. The patient had a history of type 2 diabetes mellitus for 1 year. He had no travel experience before the onset of diarrhea. No ongoing epidemic of *Escherichia coli* enteritis was reported in his county. On examination, he was afebrile with an arterial blood pressure of 154/80 mmHg. The patient was disorientated, responded slowly and improperly to questions, and was unable to move according to commands. He could not recall his food for the last meal. Other neurological physical examinations were unremarkable. Pitting edema of lower extremities was noted.

Initial investigations at admission revealed hemoglobin 127 g/L, total bilirubin 50.9 μmol/L, conjugated bilirubin 15.9 μmol/L, and serum creatinine 206 μmol/L. Urinalysis showed hematuria (+++), and protein (+++).24-h urine protein was 3.8 g, with serum albumin of 26 g/L. Lumbar puncture was performed. Cerebrospinal fluid (CSF) was colorless and transparent, with normal pressure (130mmH_2_O). Laboratory analysis of CSF revealed protein 2.47 g/L (reference range < 0.45 g/L), white blood cell count 1 cell/uL, while glucose and chloride were within normal ranges.

**Fig. 1:**
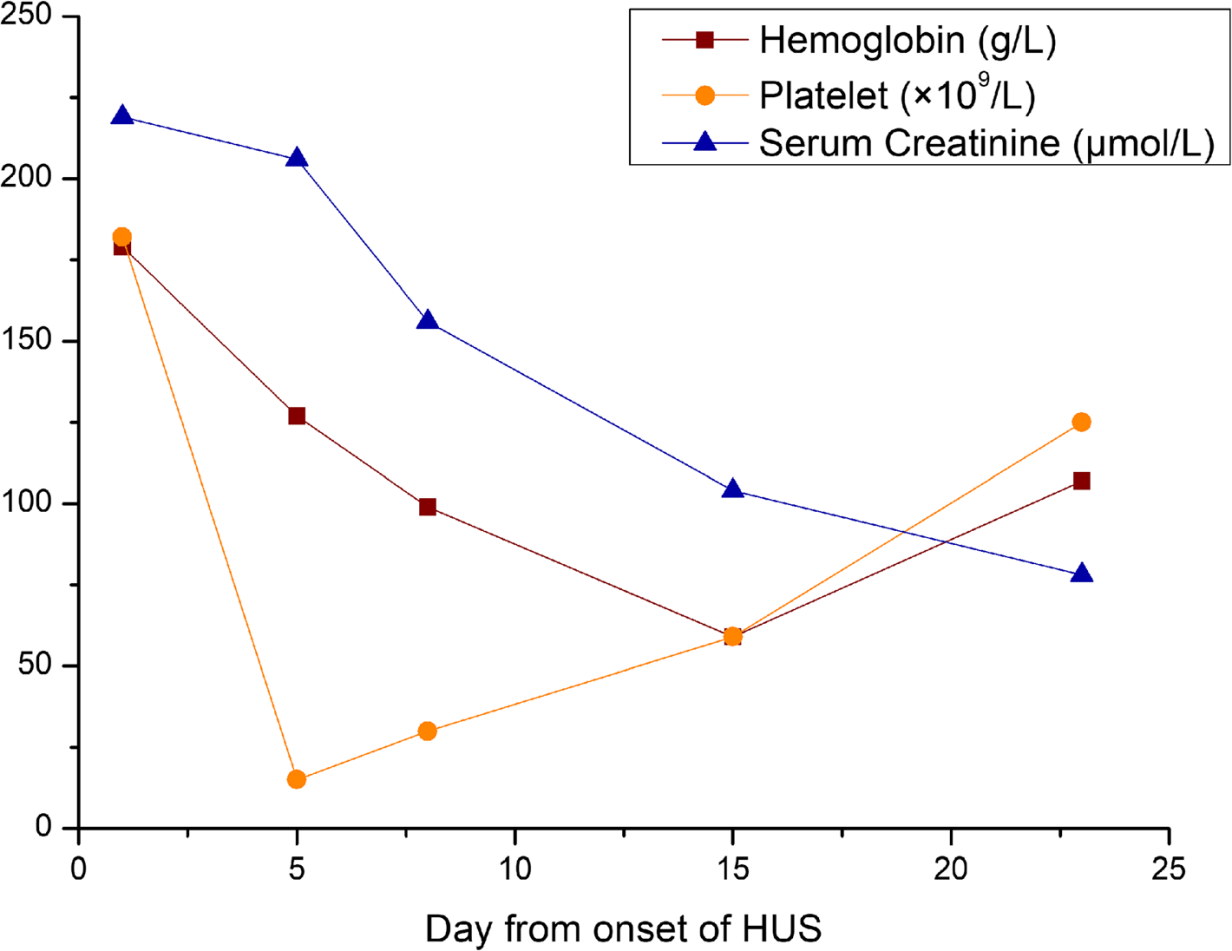
Laboratory findings. Hemoglobin (red), platelet (orange), and serum creatinine (blue) levels measured 1 to 23 days from the onset of HUS

During the first week of his hospital stay, a rapid decline of hemoglobin and platelet levels was observed (Fig. 1). Further hematological laboratory tests demonstrated elevated lactate dehydrogenase (767 unit/L) and free serum hemoglobin (12.3 mg/dL). Schistocytes were recognized on the peripheral blood smear.

**Fig. 2:**
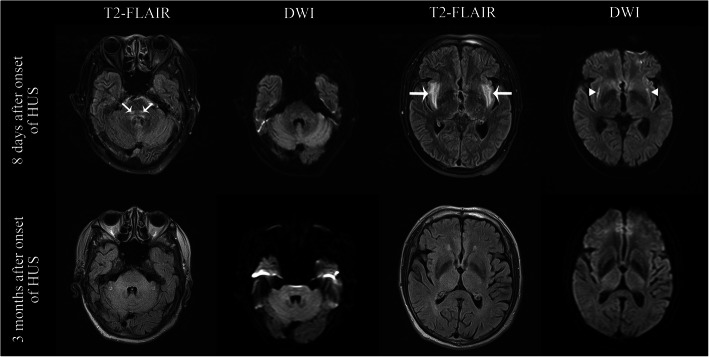
Cranial Magnetic Resonance Imaging (MRI). Bilateral hyperintensities were observed in dorsal brainstem, insula, and external capsule (arrows) on T2 weighted fluid-attenuated inversion recovery (T2-FLAIR) 8 days after onset of confusion (upper panel). The lesions within external capsule also displayed hyperintensities on diffusion weighted imaging (DWI) (triangles). Signal alterations normalized on MRI performed 3 months later (lower panel)

Cranial magnetic resonance imaging (MRI) conducted 8 days after the onset of neurologic symptoms identified symmetrical long T2 signal in the dorsal brainstem, insula, and external capsule (Fig. 2). These lesions showed restricted diffusion on diffusion-weighted imaging, and corresponding decreased apparent diffusion coefficient values. Multiple cotton-wool spots were found bilaterally on the ophthalmoscopic examination, consistent with Purtscher-like retinopathy.

Possible causes for thrombotic microangiopathy (TMA) were evaluated. Complement components C3 and C4 levels were 0.724 g/L (reference range 0.73–1.46 g/L) and 0.138 g/L (reference range 0.100–0.400 g/L), respectively. Immunological studies for antinuclear, antiphospholipid, and antineutrophil cytoplasmic antibodies were negative. ADAMTS13 activity was intact without ADMTS13 inhibitor detected. Serum concentration of complement factor H was within normal range, and anti-factor H antibody was not detected.

The patient was clinically diagnosed with post-diarrheal HUS. Initially, he received plasma exchange with fresh frozen plasma for 3 days. Renal replacement therapy was not indicated. Consciousness and cognitive functions became normal within 3 days. Hemoglobin, platelet count, bilirubin, and lactate dehydrogenase levels rapidly improved (Fig. 1), and schistocytes disappeared in blood smear within a week. Two weeks after admission, serum creatinine declined to normal range, while a repeated 24-h urine protein test revealed 2.23 g.

**Fig. 3:**
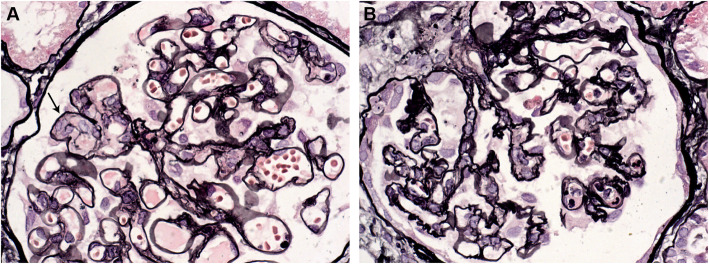
Renal pathology on light microscopy (Jones silver stain,× 400). **a** Mild mesangial hypercellularity with segmental glomerular endothelial swelling and proliferation [3]. **b** Ischemic retraction of a glomerulus with corrugation of glomerular basement membrane (GBM)

Kidney biopsy was performed 1 month after the onset of the disease to exclude other etiologies for nephrotic syndrome and assess the severity of renal damage. Renal pathology showed glomerular endothelial swelling, and segmental lamina rara interna thickening in glomerular basement membrane, without remarkable hypercellularity. Acute tubular injury was also found. (Fig. 3). Immunofluorescence staining was negative.

On a three-month follow-up, neurological sequelae were not identified. Blood pressure was 140/90 mmHg. The 24-h urine protein was 0.15 g, and previously identified central nervous system lesions were absent on the cranial MRI (Fig. 2). Self-monitored blood pressure was 130/80 mmHg 6 months after the onset without the use of antihypertensives.

## Discussion and conclusions

We reported a case presented with the classical triad of microangiopathic hemolytic anemia, thrombocytopenia, and acute kidney injury, leading to the diagnosis of TMA. The prodromal illness with bloody diarrhea and abdominal pain highly suggested STEC-HUS. The age of onset and lack of family history made hereditary causes of TMA less likely. Negative results of laboratory studies on ADAMTS13 and factor H excluded TTP and anti-factor H-antibody related HUS. Drug induced TMA should be taken into consideration. However, as gentamycin might have exacerbated renal injury by causing acute tubular necrosis, evidence of gentamycin-induced TMA was scarce, and contradicted by in-vitro studies [6, 7]. Further differential diagnosis was limited as STEC testing and genetic analysis of atypical HUS-related genes were not performed. In conclusion, although atypical HUS could not be completed excluded, the most probable diagnosis was post-diarrheal HUS.

As is typically seen in HUS, renal impairment of our patient presented as a rapid escalation of serum creatinine, microscopic hematuria, and hypertension. Nephrotic-range proteinuria at acute stage was exceptional. Among most previous reports, a comorbid primary glomerulonephritis (e.g. IgA nephropathy, acute post-streptococcal glomerulonephritis) or an underlying systemic disease (e.g. systemic lupus erythematosus) may explain the presence of nephrotic syndrome in HUS patients [8–10]. In the present case and other reports, such comorbidities were not discovered by renal biopsies [11, 12]. Although direct damage of Shiga toxin on renal endothelium has been established as the primary pathophysiological change in STEC-HUS, emerging evidence shows the verotoxin may direct cell damage after binding to the podocytes, the key target for injury in proteinuric glomerular diseases [1]. The massive proteinuria observed in the present case further displays the complexity of the biological responses behind the development of renal impairment in post-diarrheal HUS.

It has been widely accepted that the severity of kidney failure at acute illness, measured by the presence and duration of anuria or the need for dialysis, associates with worse prognosis in HUS [13, 14]. The presence of proteinuria one-year after the onset was also proposed to have long-term prognostic value [15, 16]. Less is known about the clinical significance of proteinuria level at acute stage. In theory, massive proteinuria per se may worsen kidney function. In the present case, it was observed that initial nephrotic-range proteinuria turned negative in 3 months, and serum creatinine normalized before the disappearance of proteinuria.

CNS involvement, manifested as confusion and cognitive impairment, with abnormal signals on cranial MRI, were observed in the patient’s clinical course. In the 2011 German STEC outbreak, neurological complications were reported in 48–56% of adult patients [4, 5]. Life-threatening clinical conditions (e.g. epileptic seizures, loss of consciousness) may develop, leading to intubation and intensive care unit stay in more than 30% of cases [4]. Despite its serious nature, the outcome of neurological complications in adults was above expectations. As was in the present case, neurological symptoms and neuro-imaging alterations were usually reversible, and long-term sequelae were rarely seen [4, 5].

Due to the lack of high-quality evidence, recommended management for STEC-HUS remains supportive [17]. In practice, more aggressive approaches, including plasma exchange and anti-C5 monoclonal antibody (i.e. eculizumab) have been applied to patients with severe disease. In multi-center retrospective studies on the German outbreak, neither plasmapheresis nor eculizumab was associated with clear benefit [18, 19]. However, among selected patients with neurological involvement, early use of plasma therapy and eculizumab might improve the clinical outcome [20, 21]. We gave plasma exchange to our patient due to the presence of CNS involvement and the unavailability of eculizumab then in China.

In summary, our case report highlights the presence of nephrotic-range proteinuria in an adult post-diarrheal HUS patient. Favorable outcome was observed despite nephrotic syndrome and neurological involvement during the acute stage of the disease.

## Data Availability

All data generated or analyzed during this study are included in this published article.

## Abbreviations

CSF: Cerebrospinal fluid
HUS: Hemolytic uremic syndrome
MRI: Magnetic resonance imaging
STEC: Shiga toxin-producing *Escherichia coli*
TMA: Thrombotic microangiopathy
